# Evaluation of the surface thermal response of peripheral nerve blocks in dogs undergoing trauma or orthopedic surgery

**DOI:** 10.3389/fvets.2025.1640338

**Published:** 2025-12-02

**Authors:** Alejandro Casas-Alvarado, Daniel Mota-Rojas, Patricia Mora-Medina, Julio Martínez-Burnes, Ismael Hernández-Ávalos, Ana Zapata, C. Iván Serra Aguado, Rocío Fernández-Parra

**Affiliations:** 1 Programa de Doctorado en Ciencias de la Vida y del Medio Natural, Escuela de Doctorado, Universidad Católica de Valencia San Vicente el Mártir, Valencia, Spain; 2 Doctorado en Ciencias Biológicas y de la Salud, Universidad Autónoma Metropolitana, Mexico City, Mexico; 3 Neurophysiology of Pain, Behavior and Assessment of Welfare in Domestic Animals, DPAA, Universidad Autónoma Metropolitana (UAM), Mexico City, Mexico; 4 Facultad de Estudios Superiores Cuautitlán, Universidad Nacional Autónoma de México (UNAM), Cuautitlán Izcalli, Mexico; 5 Facultad de Medicina Veterinaria y Zootecnia, Instituto de Ecología Aplicada, Universidad Autónoma de Tamaulipas, Victoria, Mexico; 6 Clinical Pharmacology and Veterinary Anesthesia, Biological Sciences Department, FESC, Universidad Nacional Autónoma de México, Cuautitlán, Mexico; 7 Hospital Veterinario de Referencia UCV, Departamento de Medicina y Cirugía Animal, Facultad de Veterinaria y Ciencias Experimentales, Universidad Católica de Valencia San Vicente Mártir, Valencia, Spain; 8 Departamento Medicina y Cirugía Animal, Facultad de Veterinaria y Ciencias Experimentales, Universidad Católica de Valencia San Vicente Mártir, Valencia, Spain

**Keywords:** canine, pain management, local anesthetics, locoregional anesthesia, infrared thermography

## Abstract

**Introduction:**

Locoregional anesthesia using local anesthetics has been proposed as a highly selective method for perioperative acute pain management because it helps prevent the onset of noxious stimuli. However, a limitation of this technique is the possibility of nerve block failure. Infrared thermography (IRT) has been suggested as a non-invasive tool to assess the success of peripheral nerve blocks by detecting temperature changes related to vasodilation. This study aimed to evaluate the effect of peripheral nerve blocks on the superficial thermal response of limbs in dogs undergoing trauma or orthopedic surgery.

**Methods:**

A total of 26 dogs of various breeds, classified as ASA 1 or 2 and undergoing thoracic or pelvic limb, or abdominal surgery, were divided into two groups based on the analgesic technique used. In the experimental group [peripheral nerve block (PNB) *n* = 20], composed of animals undergoing trauma or orthopedic surgery, bupivacaine was infiltrated into the brachial plexus or the saphenous and sciatic nerves. The control group (*n* = 6) underwent general anesthesia and surgery, and they received conventional injectable analgesia. The variables assessed included maximum (T_max_), mean (T_mean_), and minimum (T_min._) temperatures of the axillary region, groin, and lateral femoral area, as well as rectal temperature (T°C). Measurements were taken at baseline (T_Basal_), and 5 (T_5min._), 10 (T_10min._), and 15 min (T_15min._) after treatment.

**Results:**

T_max_, T_mean_, and T_min_ were significantly higher in the PNB group (by 2–3 °C) compared to the control group (*p* = 0.01). In the PNB group, superficial temperatures decreased by approximately 1 °C from baseline (*p* = 0.001), whereas the control group exhibited a greater decrease of approximately 3 °C at the same time points (*p* = 0.001). Rectal temperature was 2 °C higher in the PNB group compared to the control group (*p* = 0.01), although only the control group showed a progressive decrease over time (*p* = 0.05). No significant correlation was found between surface and rectal temperatures.

**Discussion:**

Peripheral nerve blocks with bupivacaine induced localized vasodilation, resulting in increased superficial heat radiation. This thermal response may serve as an indirect indicator complementary of nerve block effectiveness, supporting the use of IRT as a clinical tool to evaluate peripheral nerve block success in dogs. Further studies are recommended to confirm and validate its clinical application.

## 1 Introduction

Peripheral nerve block techniques are frequently used in dogs and cats to manage acute pain resulting from surgical procedures involving limb interventions (1–3). One of the primary benefits of peripheral nerve blocks is the reduction of opioid and alpha-2 adrenergic agonist use perioperatively, especially in cases of joint and long bone injuries (4). Additionally, they may help prevent the development of peripheral or central sensitization processes that can lead to hyperalgesia and allodynia in animals (5, 6). Furthermore, implementing these techniques can contribute to decreased costs related to inhalation anesthetics (7, 8). The study by Romano et al. (9) demonstrated the effectiveness of peripheral nerve blocks in reducing physiological responses to surgical stress, thereby lowering the risk of adverse effects during the surgical anesthetic procedure. Several narrative and systematic reviews in human medicine suggest that peripheral nerve blocks provide significant benefits in trauma care, as they help mitigate complications associated with acute pain, shorten hospital stays, and reduce opioid consumption (10, 11). Similarly, Entezari et al. (12) suggested that the use of peripheral nerve blocks in patients with ankle fractures reduced opioid requirements by 30% within the first 24 h postoperatively. These findings suggest both physiological and economic advantages of this analgesic strategy in surgical settings.

The use of ultrasound-guided techniques and nerve stimulation increases the chances of successfully blocking the target nerve (13–16). However, a significant limitation of these techniques is the need for specialized equipment and expertise, which involves a steep learning curve. For example, Sanranteas et al. (17) suggest that one of the reasons for failure is incorrect peripheral nerve blockade, often due to a lack of anatomical knowledge or the improper use of methods to evaluate the quality of the blockade. Researchers have proposed various assessment methods to verify the effectiveness of peripheral nerve blockade, including checking reflex responses, evaluating sensitivity to thermal stimuli, and using plethysmographic waveform changes as indicators of successful blockade (18, 19). These assessments could potentially affect the success of pain management in animals undergoing surgeries.

An alternative method to verify the effectiveness of the peripheral nerve blockade technique is the use of infrared thermography (IRT), which records changes in heat radiation associated with alterations in the local vasomotor response (20, 21). This technique involves a specialized chamber that evaluates the superficial thermal response resulting from vascular level changes that modify heat exchange with the environment due to vasoconstriction and vasodilation mechanisms that facilitate heat gain or loss (22). This phenomenon can also be attributed to the abundance of superficial blood capillaries, arteriovenous anastomoses, and hairless areas in these regions, which facilitate heat exchange with the environment (21, 23–25). Consequently, the ocular region, axilla, and plantar surface of the limbs have been identified as potential thermal windows (26, 27). In animals, the ocular region has been proposed as a reliable site for detecting changes in body temperature and for the early recognition of febrile processes in patients with infectious diseases (28–30). Moreover, since this technique detects circulatory changes, IRT can be applied to identify alterations in the surface thermal response associated with vasodilation induced by local anesthetics (31). In human medicine, it has been shown that applying IRT allows prediction of the success of the infraclavicular block by observing increases in skin temperature at the second and fifth digits (32). Similarly, Murphy et al. (33) evaluated the effect of spinal anesthesia in patients undergoing obstetric surgery, noting that spinal anesthesia caused an increase of about 2 °C in the superficial temperature of the plantar region of the foot. These changes in the surface thermal response induced by local anesthetics are proposed as indicators of the blockade’s effectiveness.

In veterinary medicine, IRT has been used to evaluate the effectiveness of dogs’ femoral-sciatic (34) and epidural nerve blocks (35). However, due to the presence of hair, selecting the correct thermal window for evaluation is essential. For instance, Küls et al. (34) did not observe significant temperature changes in the plantar region, as dogs have limited superficial circulation in that area. In contrast, Xu et al. (35) observed that epidural administration of bupivacaine in mice produced a progressive decline in the surface temperature of the pelvic limbs. These results indicate that, as in human medicine, IRT may serve as a valuable tool for evaluating the effectiveness of peripheral nerve blocks in animals. The pharmacological mechanism of local anesthetics involves the blockade of sympathetic fibers, which can modify the superficial thermal response of the pelvic limbs (36). However, previous research has not explored the potential use of areas devoid of collagen, such as the femoral and perianal regions. Some anatomical regions, known as thermal windows, have been proposed as alternative sites for temperature assessment (37). The axillary region has been identified as a possible thermal window for monitoring surface temperature changes, as suggested by Casas-Alvarado et al. (26).

Finally, it is important to note that the effectiveness of the blockade is critical for pain management, as a successful technique prevents or reduces nociception during surgery and decreases post-surgical pain. For this reason, the present study aimed to evaluate the superficial thermal response after limb peripheral nerve blocks in dogs undergoing trauma surgery. We hypothesize that administering bupivacaine around peripheral nerves induces an increase in heat radiation compared to control animals.

## 2 Materials and methods

### 2.1 Animals and experimental design

This prospective clinical study aimed to replicate conditions in a real intra-teaching hospital environment (38). It was conducted between September 2023 and January 2024 at the Veterinary Teaching Hospital of the Catholic University of Valencia. The study was evaluated and approved by the Ethics Committee of the Catholic University of Valencia, with registration number CEEAUCV 2306, under the owners’ informed consent for all animals. Additionally, the study was conducted in strict accordance with ethical guidelines for the use of client-owned animals in clinical research and was carried out in compliance with the ethical principles and regulatory standards established by the Veterinary Teaching Hospital the Catholic University of Valencia (39).

For this study, dogs scheduled for orthopedic surgery at our veterinary referral teaching hospital were potentially selected to be included in the treatment group. In contrast, animals in the control group consisted of those scheduled for general anesthesia, where injectable analgesia was administered during procedures that did not require peripheral nerve blockade. According to the American Society of Anesthesiologists, all dogs underwent a complete general physical examination, blood cell count, and serum biochemistry to determine the anesthetic risk. Dogs classified as ASA-1 (healthy animals) and ASA-2 (animals with mild systemic disease) were included in the study. Animals classified as ASA-3 or higher and those with severe infectious disease, acute renal disease (IRIS 2 or 3), coagulation disorders, and animals less than 1 year old were excluded from the study.

**Figure 1:**
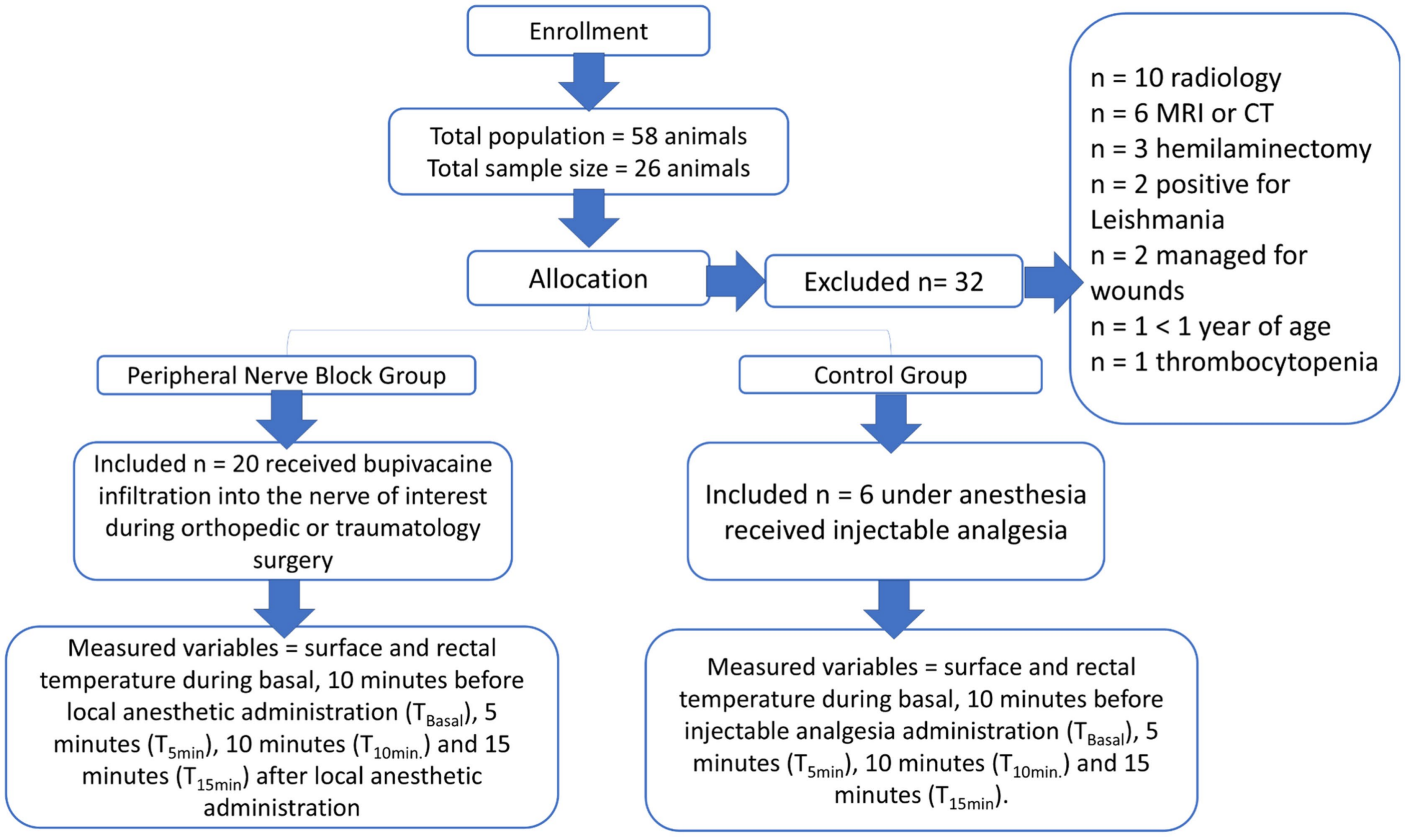
Flow diagram of animals throughout phases of the study. CT (Computed Tomography), MRI (Magnetic Resonance Imaging).

A total of 26 animals, representing both sexes, were allocated into two groups based on clinical indications and their corresponding treatments. The peripheral nerve block group (PNB, *n* = 20) received a 0.25% bupivacaine injection targeted at the relevant nerves during traumatic or orthopedic surgery (involving the brachial plexus or saphenous and sciatic nerves). The control group (control, *n* = 6) consisted of animals under general anesthesia that received injectable analgesia (40). The IRT and rectal temperature variables were measured and recorded at the following time points: baseline; 10 min before local anesthetic administration (T_Basal_); 5 min (T_5min._), 10 min (T_10min._), and 15 min (T_15min._) after local anesthetic administration in the PNB group; and after surgical clipping, scrubbing, and 10 min after receiving injectable analgesia in the control group (Figure 1).

### 2.2 Anesthesia management and peripheral blocks

Animals were fasted for 12 h with water until 2 h before surgery. Premedication was performed intramuscularly (IM) with medetomidine at 10 μg kg^−1^ (Medetor, Virbac, Spain) and methadone at 0.2 mg Kg^−1^ (Semfortan, Dechra, Spain), combined in the same syringe and injected into the *cleidocephalicus* muscle. Once sedation was achieved, all animals were aseptically catheterized with an intravenous catheter in either the cephalic or saphenous vein (Introcan®, B. Braun VetCare, Germany). Lactated Ringer’s solution (Lactato-RingerVet, B. Braun VetCare, Germany) was infused at a rate of 5 mL kg^−1^ h^−1^ throughout the surgical procedure.

After pre-oxygenation, anesthetic co-induction was performed to effect with intravenous (IV) administration of ketamine at 1 mg kg^−1^ (Ketamidor, Girovet, Spain) and propofol (Propovet, Richmond VetPharma, Spain) at 2–4 mg Kg^−1^ to effect. Once unconsciousness was achieved, orotracheal intubation was performed, and the animals were subsequently connected to a rebreathing anesthetic circuit (Smiths Medical Surgivet, Pigeon, UK) with an oxygen flow of 50 mL kg^−1^ min^−1^ for the first 20 min, then reduced to 25 mL kg^- 1^ min^−1^. Maintenance of anesthesia was carried out through the administration of isoflurane (Isoflutek; Karizoo, Spain) vaporized in a 50–60% oxygen-medical air–gas mixture.

After induction of anesthesia, clipping, and skin preparation, an ultrasound-guided peripheral nerve block was performed with a linear probe ML6-15 (Logiq P9, General Electric, Japan) and an echogenic stimulating needle (Stimuplex® Ultra 360®, B. Braun, Germany). In case of thoracic limb surgery, a subscalenic brachial plexus block was performed (41) with 1 mg kg^−1^ obtained from a dilution with a solution containing 0.25% bupivacaine (Bupivacaine 5 mg ml^−1^, B. Braun, Germany). For the pelvic limb, a saphenous nerve block (14) and a sciatic nerve block (1) were conducted using 0.5 mg Kg^−1^ of 0.25% bupivacaine solution per nerve, resulting in a total dose of 1 mg kg^−1^, as administered in the PNB group.

Ventilation was initiated under volume-controlled mechanical ventilation (Aespire View, GE Healthcare, USA) in patients deemed obese, those experiencing apnea after induction, or patients with RR less than 6 rpm, moderate hypercapnia (pCO_2_ > 55 mmHg), or end-tidal carbon dioxide (ETCO_2_) values higher than 55–60 mmHg. Adjustment settings were made to sustain an ETCO_2_ between 35 and 45 mmHg. The vaporizer was adjusted to achieve an end-expiratory fraction of isoflurane (Fe′Iso) between 1.0 and 1.2% prior to starting surgery and was then adapted to maintain a Plane II anesthesia (no palpebral reflex, ventromedial ocular globe, and no mandibular tone). The mean percentage of Fe′Iso used was calculated for each group. The same anesthesiologist (AZ) conducted and oversaw the entire anesthetic procedure.

### 2.3 Rescue analgesia

Rescue analgesia consisted of a bolus of fentanyl at a dosage of 2 μg Kg^−1^ intravenously. It was provided when there is an increase by more than 20% in heart rate and systolic blood pressure and/or when there is a rise of more than 20% in respiratory rate or movement of the animal following a nociceptive surgical stimulus (42). Fentanyl consumption by total use of μg Kg^−1^ administered in each case was calculated.

### 2.4 Infrared thermography images acquisition and analysis

**Figure 2:**
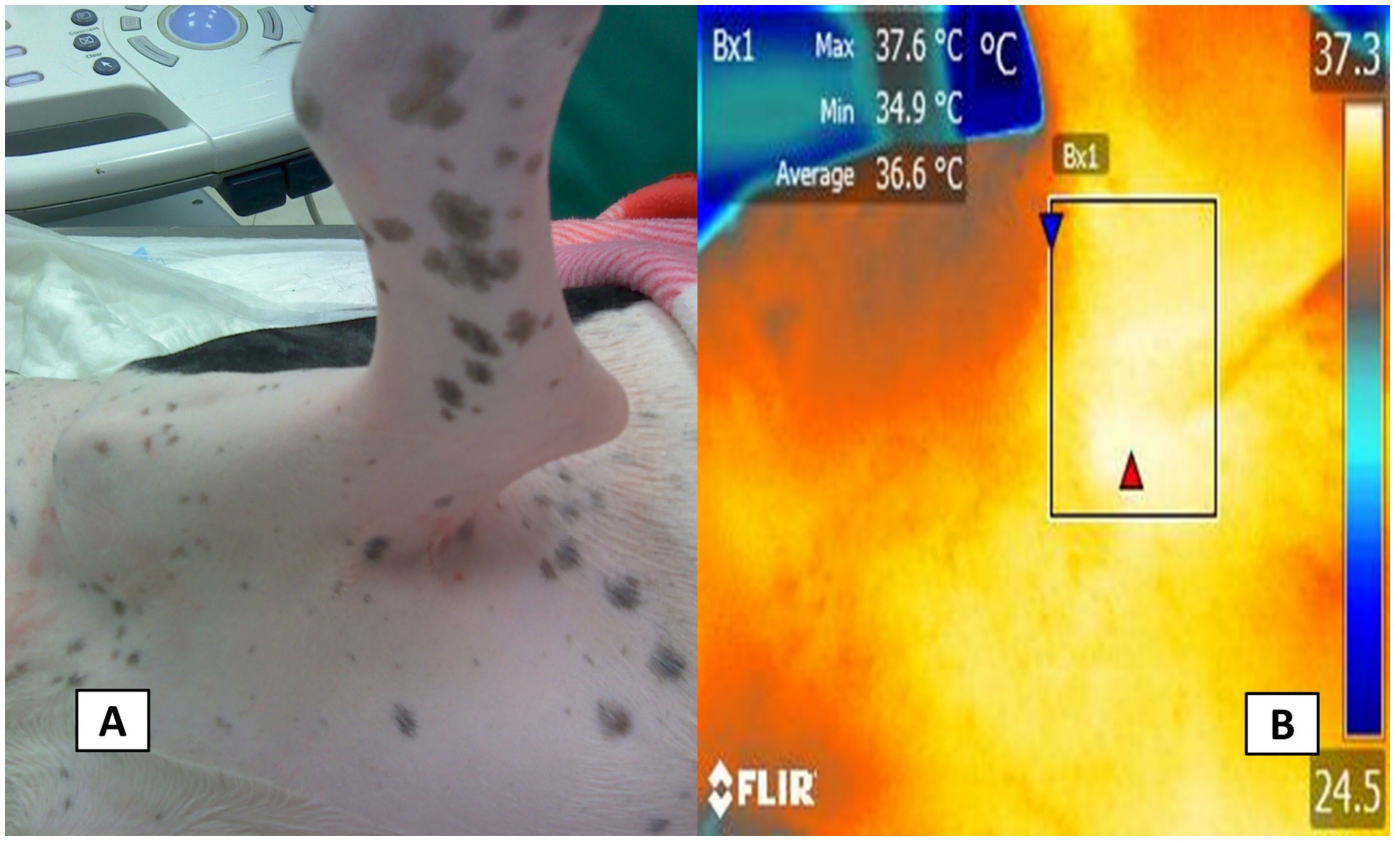
Thermal window evaluated in the thoracic limb. The digital image **(A)** of the radiometric image capture of the axillary thermal window in projection to the axillary space of the thoracic limb is shown **B**. Axillary thermal window. In a projection of the axillary region (Bx1) of the thoracic limb, a rectangle of 3-cm long × 2-cm wide was used to obtain the irradiated surface temperature coming from the brachial artery that irrigates the same brachial plexus.

**Figure 3:**
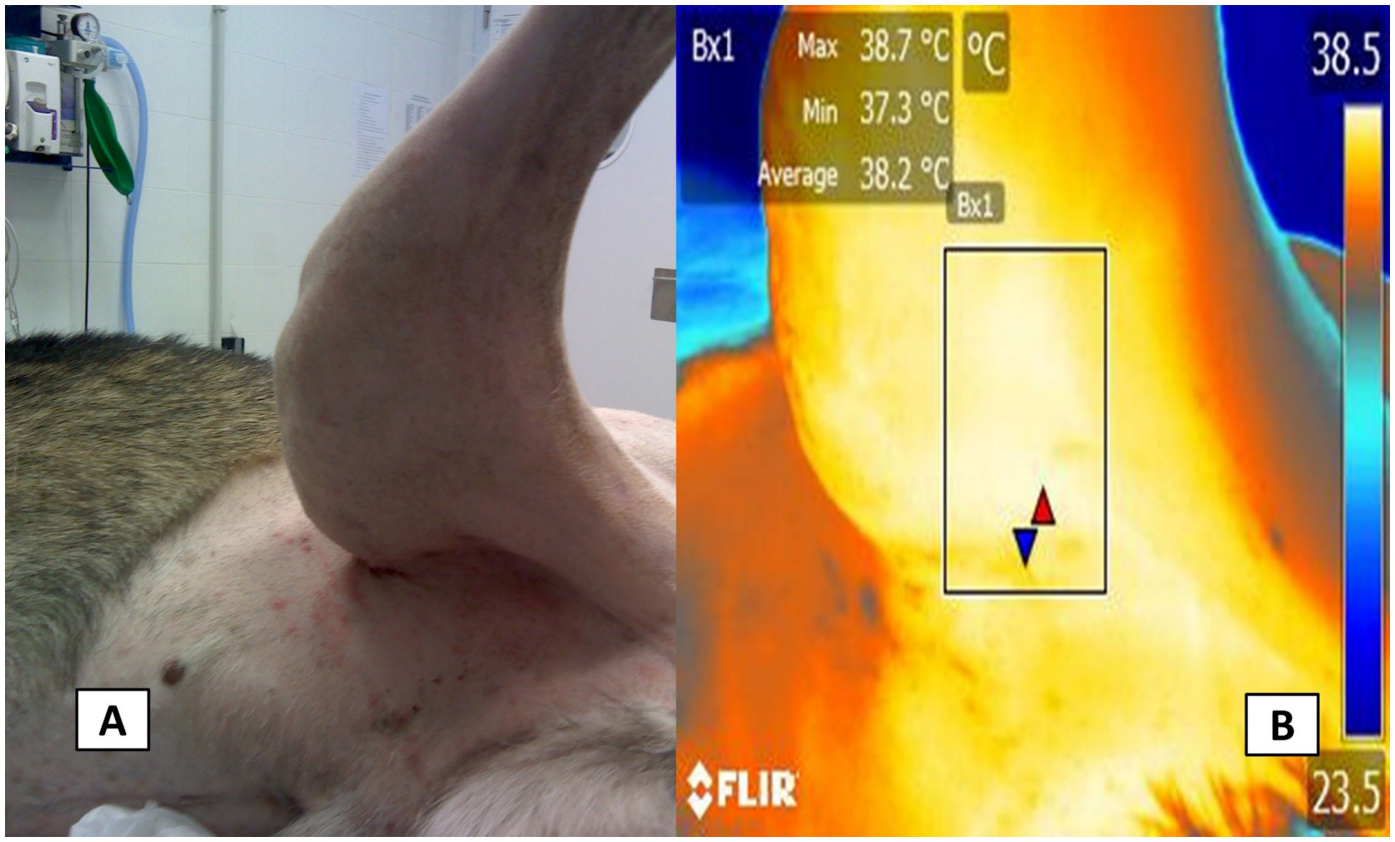
Thermal window evaluated in the pelvic limb. The digital image **(A)** of the radiometric image capture of the femoral thermal window in projection to the inguinal space of the pelvic limb is shown **B**. Femoral thermal window. With a radiometric image in the projection of the inguinal region of the pelvic limb, a rectangle of 3-cm long × 2-cm wide was used to obtain the irradiated surface temperature coming from the femoral artery that irrigates the same saphenous nerve block.

**Figure 4:**
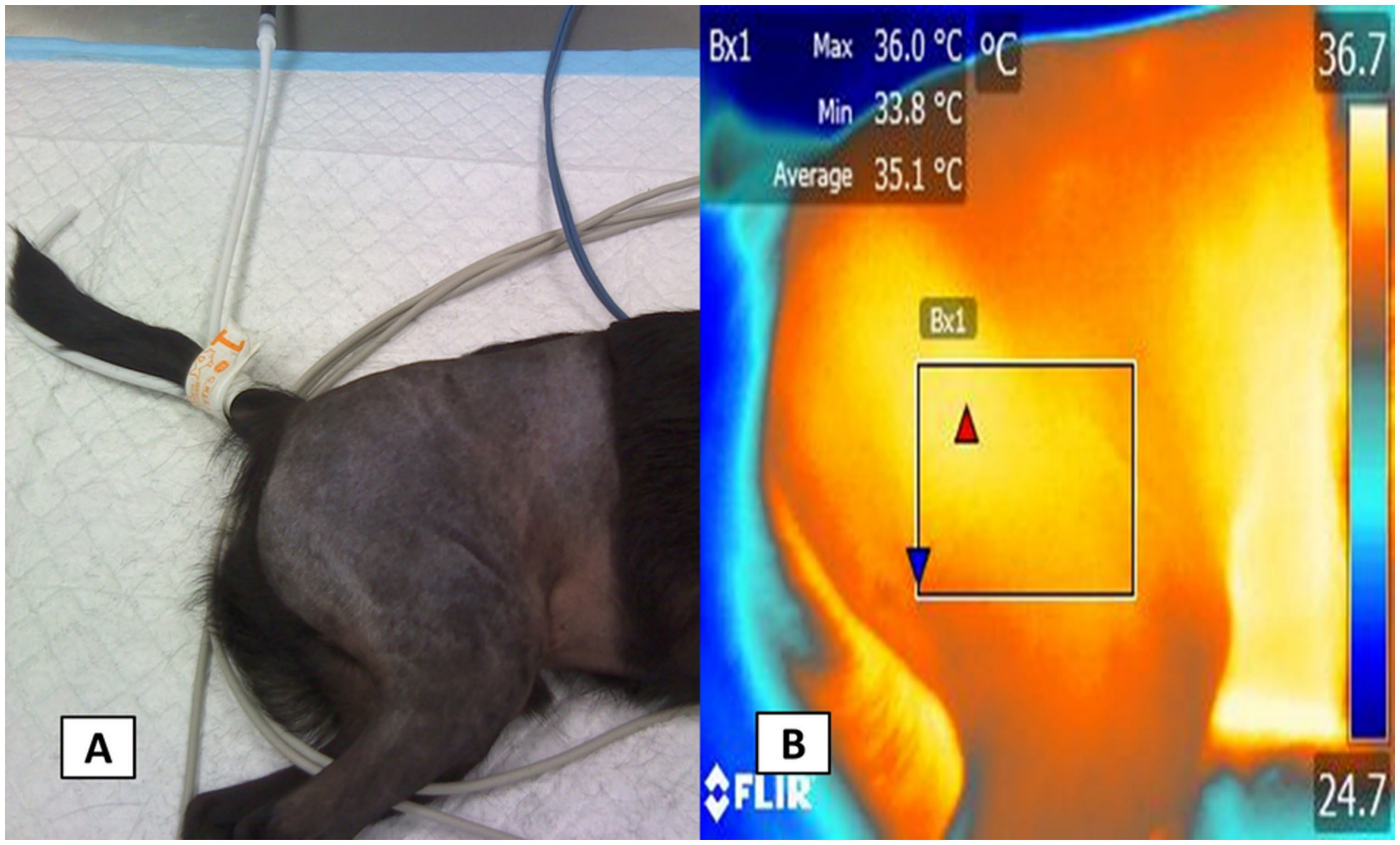
Thermal window evaluated in pelvic limb in projection lateral. The digital imagen **(A)** of the radiometric image capture of the sciatic thermal window in projection to the lateral in the pelvic limb is shown **B**. The lateral sciatic thermal window. For this region, a radiometric image of the femoral region was obtained in a lateral projection that allowed the evaluation of the surface temperature of the femoral artery that allows the irrigation of the sciatic nerve. It should be noted that the maximum (red triangle), mean and minimum (blue triangle) surface temperature was obtained in all the radiometric images.

Radiometric images were obtained using a FLIR Thermal TM E580 infrared camera (FLIR Systems, USA). The collected images were captured at 1-m distance with a resolution of 320 × 240 pixels from the clipped skin of the area of interest and had an emissivity of 0.95 in a room with an average temperature of 25 °C and controlled humidity of 40%. In the case of the thoracic limb, the axillary region was used when the brachial plexus block was indicated (Figure 2A). The image for the saphenous nerve block was captured in the projection of the inguinal region (Figure 3A) while for the sciatic block, an image in the lateral projection of the mid-thigh region was obtained (Figure 4). Additionally, the rectal temperature was recorded using a digital rectal thermometer (model DMT-4233, COMPANY, China).

The acquired images were then imported into FLIR Tools® software (FLIR Systems, USA) for radiometric image analysis, during which regions of interest were manually outlined with a box measuring approximately 3 cm in length and 2 cm in width. A researcher trained in evaluating radiometric images and unaware of the animal’s treatment carried out the image analysis. For the axillary region, shown in Figure 2A, the box was drawn to include the heat emitted from the medial side of the axillary artery. Similarly, in the projection of the inguinal region (Figure 3B), a box was placed to capture heat from the femoral artery. Furthermore, for the lateral view of the sciatic block, a box was delineated to record heat from the branches of the caudal gluteal artery and lateral circumflex femoral arteries (Figure 4B) (26). These thermal windows allowed for the measurement of maximum surface temperature (T_max_), mean temperature (T_mean_), and minimum temperature (T_min_) within the respective regions of interest.

### 2.5 Statistical analyses

The sample size was determined using G*power 3.1.9.7 software (Heinrich-Heine-Universität Düsseldorf, Düsseldorf, Germany). Based on the software’s calculations and the previous mean and standard deviation from a pilot study conducted by Casas-Alvarado et al. (43), a minimum of 10 animals in the treatment group were estimated to detect a 1 °C temperature difference between the treatment and control group skin surfaces. We considered an error probability (*α*) of 0.05, a 95% confidence level, a power of 0.95 (1-α), and a correction factor of 0.5 for repeated measures across the two experimental groups.

The analysis of all the data was performed using the GraphPad Prism statistical package (Ver. 9.5.1, Dotmatics, USA), where the arithmetic mean with its standard deviations (SD) was obtained for the variables of infrared thermography and rectal temperature in the PNB and control groups during all the evaluation times (T_Basal_, T_5min._, T_10min._, _and_ T_15min._). Under these conditions, an analysis of sphericity and residuals was conducted. The parametric variables (thermography and rectal temperature) were subjected to analysis of variance (ANOVA) using a linear mixed model under the specified conditions.


Yijak=μ+τi+τj+τiτj+βk+eij

Where: Y = Response variable (surface temperature and rectal temperature); τi = Treatment effect; τj = Event effect; β = Random effect (animal); μ = Population average; e = Residual.

A Tukey’s *post hoc* test was used to evaluate differences between the mean values. Finally, the total consumption of opioids and the total expenditure of inhalation anesthetics were analyzed using one-way variance analysis. In all cases, a significance level of *p* < 0.05 was set. Correlations between variables were determined using Pearson’s correlation.

## 3 Results

The period covered by this study was from September 2023 to January 2024. A total of 58 cases were obtained; however, only 26 client-owned dogs were finally included (Figure 1). The dogs had a mean age of 3 ± 1.5 years and a range body condition score 3 – 4. They were divided into PNB, *n* = 20 and control, *n* = 6 groups. The distribution of breeds in the groups was as follows: the PNB group included 4 Gos Rater Valencià, 2 Shiba Inu, 4 Poodles, 1 Dachshund, 1 Yorkshire Terrier, 1 Shar Pei, and 6 mixed-breed dogs. In this group, 12 dogs were males and 8 were females. The control group included 2 Belgian Shepherds, 2 Labradors, 1 Pyrenean Mastiff, and 1 Poodle. This group included 5 males and 1 female (Table 1). We excluded 10 dogs that underwent imaging (radiology), 6 dogs that underwent advanced imaging (MRI or CT), 3 dogs that had hemilaminectomy surgery, 2 dogs that tested positive for leishmaniasis, 2 dogs receiving wound management, 1 dog under 1 year of age, and 1 dog with thrombocytopenia (Figure 1).

**Table 1:** Baseline characteristics of the animals included in the study.

Breed	Weight (Kg)	Type surgery	Block performed	Baseline temperature (° C)
Belgian Shepherds	12.5	Radical mastectomy	Without block	38.1
Belgian Shepherds	14.1	Subcutaneous ureteral bypass	Without block	38.8
Poodle	8.9	Subcutaneous ureteral bypass	Without block	38.6
Labrador	32.8	Hip replacement surgery	Without block	38.7
Labrador	48	Hip replacement surgery	Without block	38.5
Pyrenean Mastiff	46.7	Hip replacement surgery	Without block	38.6
Shiba Inu	12.2	Tibial plateau leveling osteotomy	Sciatic and saphenous nerves	37.6
Shiba Inu	13.4	Tibial plateau leveling osteotomy	Sciatic and saphenous nerves	38.2
Gos Rater Valencià	4.5	Osteosynthesis surgery for fracture of the tibial	Sciatic and saphenous nerves	37.6
Gos Rater Valencià	5.6	Osteosynthesis surgery for fracture of the femur	Sciatic and saphenous nerves	38.2
Gos Rater Valencià	6.2	Osteosynthesis surgery for fracture at the level of the distal radius	Brachial plexus	37.6
Gos Rater Valencià	5.5	Osteosynthesis surgery for fracture at the level of the distal radius	Brachial plexus	37.8
Poodle	7.2	Osteosynthesis surgery for fracture at the level of the proximal radius	Brachial plexus	39
Poodle	6.5	Osteosynthesis surgery for fracture of the femur	Sciatic and saphenous nerves	39.4
Poodle	5.8	Osteosynthesis surgery for fracture of the tibial	Sciatic and saphenous nerves	38.3
Poodle	6.8	Osteosynthesis surgery for fracture at the level of the distal radius	Brachial plexus	37.7
Yorkshire Terrier	3.2	Osteosynthesis surgery for fracture at the level of the proximal radius	Brachial plexus	38.8
Dachshund	9.2	Osteosynthesis surgery for fracture at the level of the distal radius	Brachial plexus	38.8
Shar Pei	24.5	Osteosynthesis surgery for fracture at the level of the proximal radius	Brachial plexus	38.7
Mixed breed	14.2	Osteosynthesis surgery for fracture at the level of the distal radius	Brachial plexus	39
Mixed breed	15.6	Osteosynthesis surgery for fracture of the femur	Sciatic and saphenous nerves	37
Mixed breed	16.5	Osteosynthesis surgery for fracture of the femur	Sciatic and saphenous nerves	39
Mixed breed	12.3	Osteosynthesis surgery for fracture at the level of the proximal radius	Brachial plexus	38.3
Mixed breed	10.8	Osteosynthesis surgery for fracture of the femur	Sciatic and saphenous nerves	37
Mixed breed	22	Osteosynthesis surgery for fracture of the femur	Sciatic and saphenous nerves	38.5

A total of 176 radiometric images of the thermal windows were captured in the study from 12 dogs that underwent brachial plexus block, 11 that underwent saphenous nerve block, and 4 that underwent sciatic nerve block. The study population in which the block was performed comprised 20 animals: 12 dogs that underwent osteosynthesis for femoral or tibial fractures in the pelvic limb or distal or proximal radius fractures in the thoracic limb, and 2 dogs that underwent tibial plateau leveling osteotomy. In addition, 6 dogs were included that underwent arthroscopy. The control group consisted of 6 animals: 3 dogs subjected to hip replacement surgery, 2 dogs that underwent subcutaneous urethral bypass, and 1 dog that underwent radical mastectomy.

**Figure 5:**
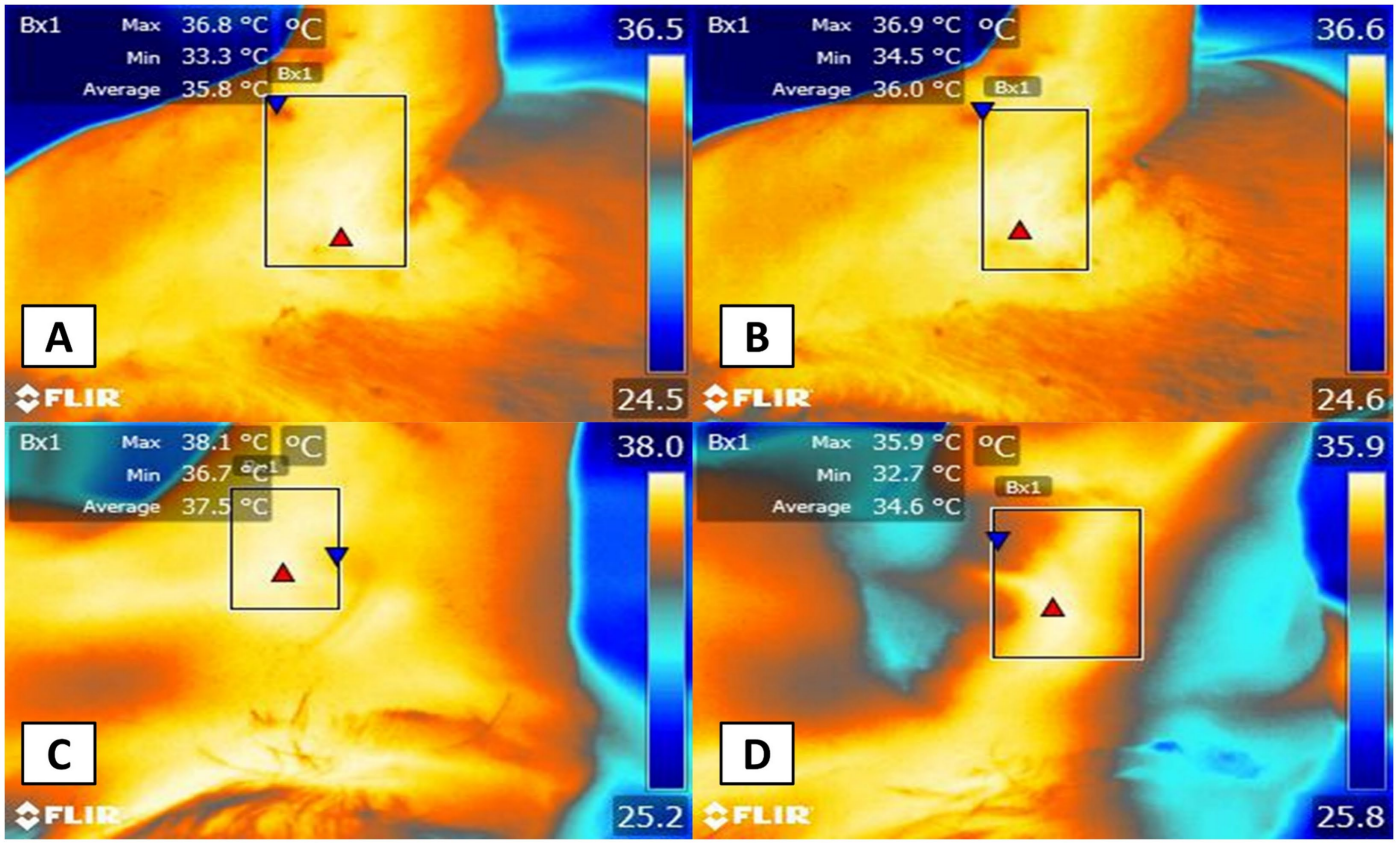
Representative thermal images from two patients, one in the peripheral nerve block (PNB) group and another in the control group are shown at baseline **(A,C)** and 5 min after the block **(B,D)**. Panels **A** and **B** display the axillary thermal window, while panels **C** and **D** show the femoral thermal window.

The surface thermal response of different regions of interest showed significant differences between evaluation times and study groups after the administration of bupivacaine. Table 2 shows the T_max_, revealing that the surface temperature of the PNB was 2.3 °C higher than the control at T_5min._ (*p* = 0.001), T_10min._ (*p* = 0.05), and T_15min._ (*p* = 0.01) as represented in Figure 5. Both groups experienced a decrease in maximum surface temperature over time. The PNB group showed significant differences at T_5min._, T_10min._, and T_15min._ compared to T_Basal_ values (*p* = 0.005, *p* = 0.002, *p* = 0.01)_._ Similarly, in the control group, there was a significant reduction in surface temperature at T_5min._, T_10min._, and T_15min._ compared to T_Basal_ (*p* = 0.001, *p* = 0.03, *p* = 0.005).

**Table 2:** Maximum surface temperature (T_Max_) of the region of interest (T °C) (Mean±SD) in dogs before and after peripheral nerve block (PNB) and control groups under general anesthesia.

Group	Temperature (°C)				*p*-value intragroup
	T_Basal_	T_5min._	T_10min._	T_15min._	
PNB (*n* = 20)	38.22 ± 0.12^1,a^	37.16 ± 0.70^1,b^	37.65 ± 0.36^1,b^	37.38 ± 0.51^1,b^	*p* = 0.003
Control (*n* = 6)	37.55 ± 0.29^1,a^	34.82 ± 0.51^2,b^	34.17 ± 0.54^2,b^	33.62 ± 0.55^2,c^	*p* = 0.001
*p*-value groups	*p* = 0.39	*p* = 0.01	*p* = 0.05	*p* = 0.01	

1,2 = significant differences between treatments groups, *p* ≤0.05 ; a,b,c = intragroup significant differences between times, *p* ≤0.05 (T_Basal_, T_5 min._, T_10min._, and T_15min._).

Table 3 shows the values of the mean surface temperature (T_mean_) values in the regions of interest. The PNB mean surface temperature was 2.6 °C, 3.6 °C, and 4.4 °C higher at T_5min._ (*p* = 0.001), T_10min._ (*p* = 0.02), and T_15min._ (*p* = 0.01) compared to control. Between events, it was observed that the surface temperature at PNB decreased significantly by 1.3 to 1.6 °C at T_5min._, T_10min._, and T_15min._, respectively, compared to T_Basal_ (*p* = 0.0006). In the control, the temperature showed a significant decrease of 2.7 °C, 3.3 °C, and 4.2 °C at T_5min._, T_10min._, and T_15min._ respectively, compared to T_Basal_ (*p* = 0.001). The temperature at T_15min._ was significantly lower than the rest of T_Basal_ (*p* = 0.001).

**Table 3:** Mean surface temperature (T_mean_) of the region of interest (T°C) (Mean±SD) in dogs before and after peripheral nerve block (PNB) and control groups under general anesthesia.

Group	Temperature (°C)				P-value Intragroup
	T_Basal_	T_5min._	T_10min._	T_15min._	
PNB (*n* = 20)	37.47 ± 0.19^1,a^	35.84 ± 1.03^1,b^	36.27 ± 0.84^1,b^	36.12 ± 0.81^1,b^	*p* = 0.0006
Control (*n* = 6)	36.83 ± 0.39^1,a^	33.23 ± 0.56^2,b^	32.63 ± 0.55^2,b^	31.77 ± 0.63^2,d^	*p* = 0.001
*p*-value group	*p* = 0.34	*p* = 0.001	*p* = 0.02	*p* = 0.01	

1,2 = significant differences between treatments groups, *p* ≤0.05 ; a,b,c = intragroup significant differences between times, *p* ≤0.05 (T_basal_, T_5 min._, T_10min._, and T_15min._).

The results of the low surface temperature (T_min_) are shown in Table 4. The PNB group presented a surface temperature between 2.6° C, 3.6° C, and 4.3° C, significantly higher at T_5 min._ (*p* = 0.03), T_10min._ (*p* = 0.001), and T_15min._ (*p* = 0.01) compared to control. In the control, there was a progressive temperature decrease, 3.1 °C, 4.3 °C, and 5.7 °C compared to T_Basal_ (*p* = 0.05). While in PNB, the surface temperature was between 1 and 0.8 °C, significantly lower than T_Basal_ (*p* = 0.008).

**Table 4:** Low surface temperature (T_min_) of the region of interest (T °C) (Mean±SD) in dogs before and after peripheral nerve block (PNB) and control groups under general anesthesia.

Group	Temperature (°C)				*p*-value intragroup
	T_Basal_	T_5min._	T_10min._	T_15min._	
PNB (*n* = 20)	35.40 ± 0.52ª^,1^	34.58 ± 1.27^b,1^	34.62 ± 1.14^b,1^	34.05 ± 1.17^b,1^	*p* = 0.008
Control (*n* = 6)	34.30 ± 0.44ª^,1^	31.18 ± 0.62^b,2^	30.02 ± 0.82^c,2^	29.14 ± 0.70^c,2^	*p* = 0.05
*p*-value group	*p* = 0.99	*p* = 0.03	*p* = 0.001	*p* = 0.01	

1,2 = significant differences between treatments groups, *p* ≤0.05 ; a,b,c = intragroup significant differences between times, *p* ≤0.05 (T_basal_, T_5min._, T_10min._, and T_15min._).

Table 5 shows the results obtained regarding rectal temperature. In the PNB, the rectal temperature was 1.6° C and 1.5° C significantly higher in the T_5min._, and T_10min._, and T_15min._ compared to the control. Between events, only the control presented a significant decrease between 1.7 and 0.9 °C compared to the T_Basal_ (*p* = 0.05). Finally, no significant correlation (*p* = 0.99) was observed between the surface temperature of the region of interest and rectal temperature.

**Table 5:** Mean rectal temperature (T ° C) (Mean±SD) in dogs before and after peripheral nerve block (PNB) and control groups under general anesthesia.

Group	Events				*p*-value intragroup
	T_Basal_	T_5min._	T_10min._	T_15min._	
PNB (*n* = 20)	38.68 ± 0.22^1,a^	39.08 ± 0.20^1,a^	38.40 ± 0.2^1,a^	38.15 ± 0.18^1,a^	*p* = 0.40
Control (*n* = 6)	38.32 ± 0.20^1,a^	37.47 ± 0.29^2,a^	36.96 ± 0.24^2,b^	36.63 ± 0.20^2,b^	*p* = 0.05
*p*-value Group	*p* = 0.82	*p* = 0.01	*p* = 0.03	*p* = 0.05	

1,2 = significant differences between treatments groups, *p* ≤0.05 ; a,b,c = intragroup significant differences between times, *p* ≤0.05 (T_basal_, T_5min._, T_10min._, and T_15min._).

**Figure 6:**
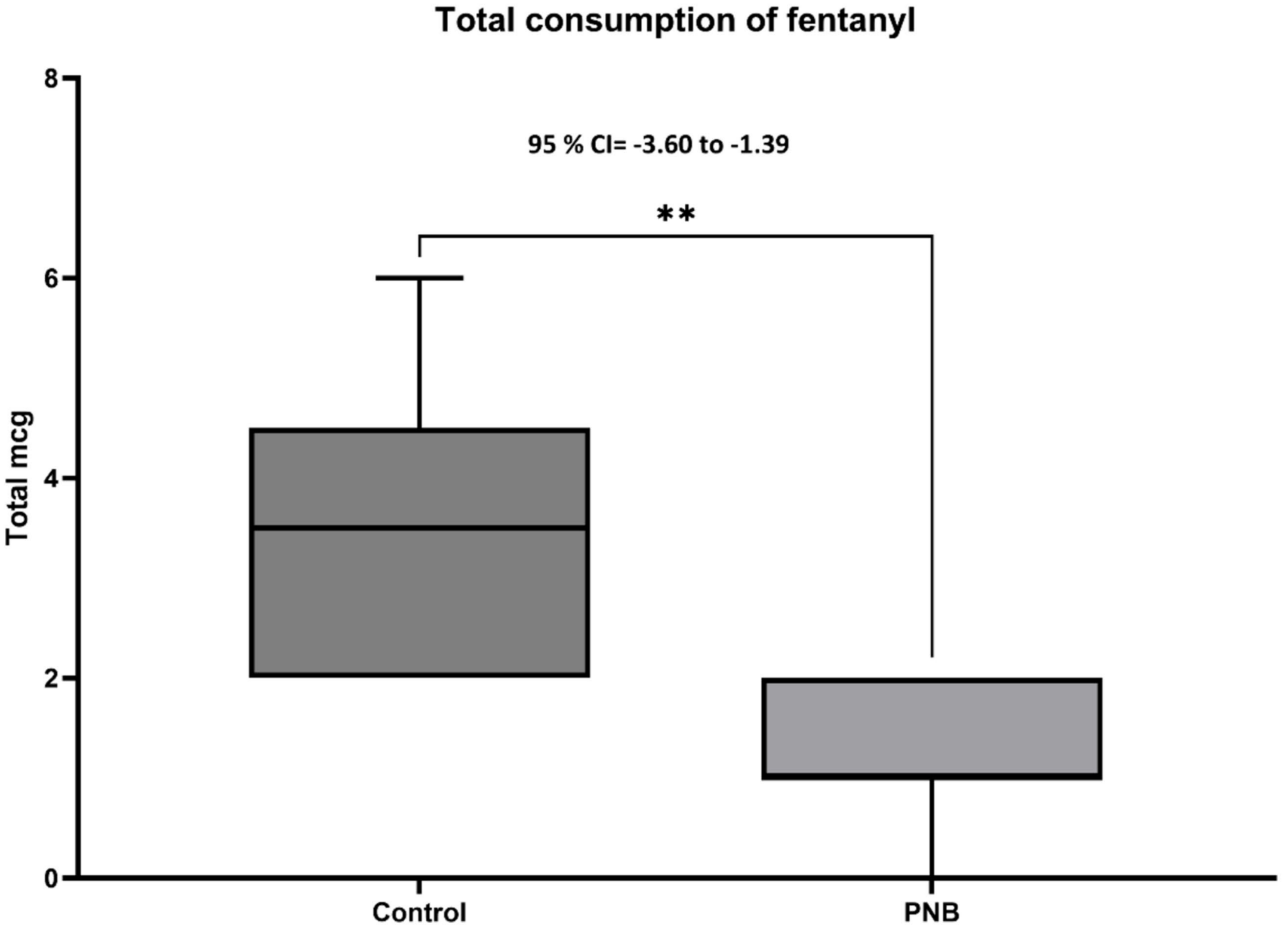
Total consumption of fentanyl (Mean±SD and 95% CI) in both groups during the procedure. Peripheral nerve block (PNB, *n* = 20) and control (*n* = 6) groups. ** Significant difference between groups.

**Figure 7:**
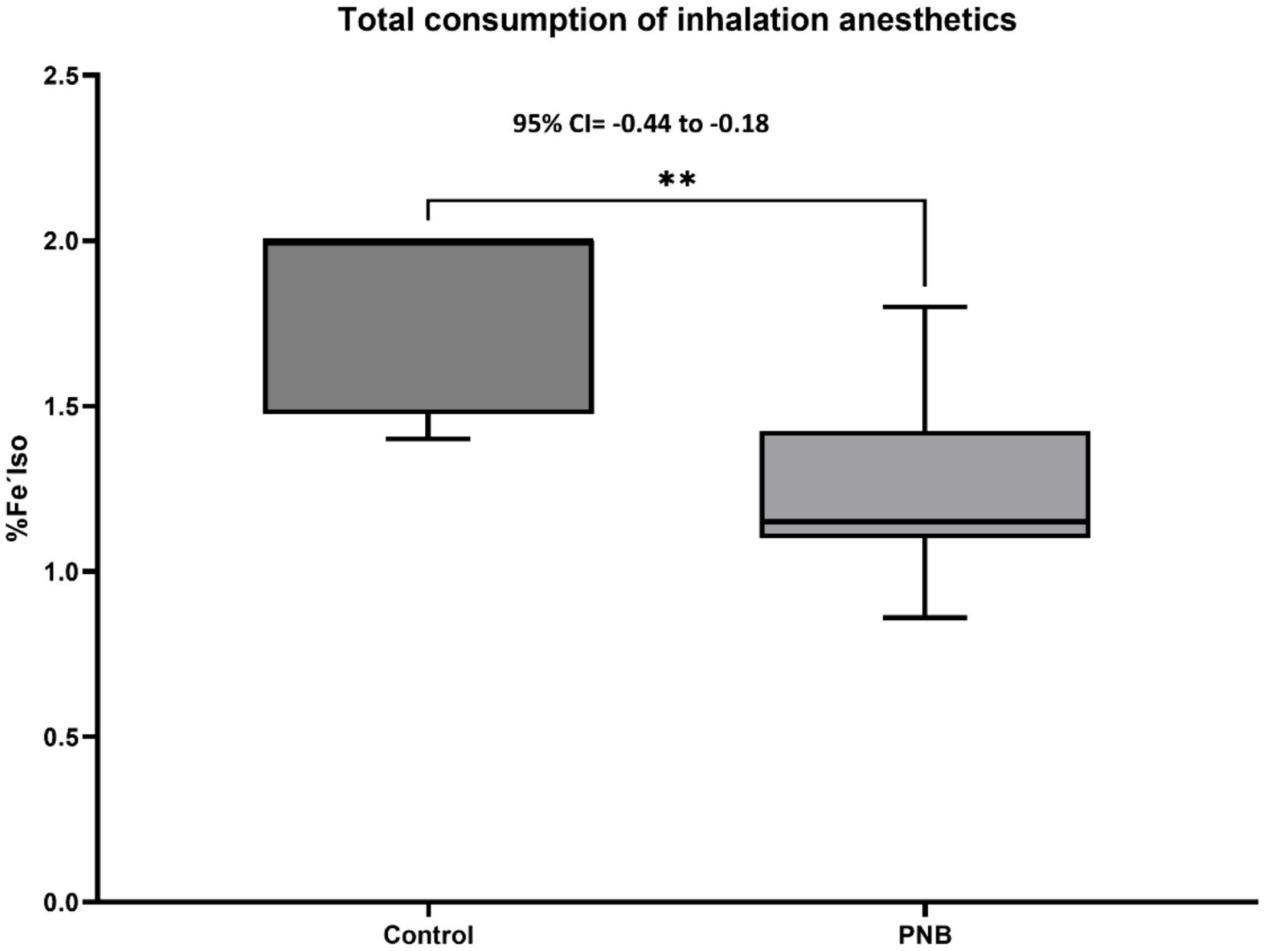
Total consumption of inhalation anesthetics (Fe′Iso%) (Mean±SD and 95% CI) in both groups under general anesthesia. Peripheral nerve block (PNB, *n* = 20) and control (*n* = 6) group. *** Significant difference between groups *p* < 0.0001.

Finally, Figure 6 shows that the total fentanyl consumption in the control group was 3.5 ± 1.5 μg Kg^−1^, which was significantly higher than in the PNB group 1.2 ± 0.7 μg Kg^−1^ (*p* = 0.003). Moreover, Figure 7 shows that the total percentage of Fe′Iso used in the control group was 1.41 ± 0.04%, which was 0.58% higher than in the PNB group 1.10 ± 0.04% (*p* = 0.0001).

## 4 Discussion

Dogs receiving local anesthetics for peripheral nerve blockade showed significantly higher the cutaneous T_max_, T_mean_, and T_min_ compared to the control group. This phenomenon can be explained by the mechanism of action of local anesthetics, which involves blocking Na^+^ channels in nociceptive Aδ and C fibers, thereby inhibiting the transduction and transmission of painful stimuli (44). Although this effect is responsible for the analgesic mechanism (45, 46), it can also occur in sympathetic B fibers and Aß and Aα fibers, which are motor fibers, leading to vasodilation and muscle relaxation (19). In other words, blocking Na^+^ ion channels may cause local vasodilation, which could result in greater heat loss due to increased heat exchange with the environment (47).

The vasodilation caused by the drugs can be directly linked to the analgesic effect. To achieve this pain relief, a combined block of the saphenous nerve and the sciatic nerve is recommended to provide analgesia for the medial and cranial areas of the stifle without affecting the motor function of the quadriceps muscle, when used together with a sciatic nerve block (1). However, it is important to note that bupivacaine has a slow onset of action (20–30 min) (48). Therefore, it is possible that when the study variables were collected in this experiment, they were primarily related to peripheral vascular effects such as vasodilation and the negative impact on thermoregulation due to increased heat loss.

The findings of this study align with observations in human medicine, where it has been demonstrated that nerve blockade at the clavicular or sciatic level induces vasodilation, resulting in increased fingertip temperatures in hands or feet (19, 32, 49, 50). This vasodilation, triggered by the blockade of sympathetic fibers, serves as the basis for alterations in blood flow, potentially influencing the surface thermal response detected by IRT (51, 52). Mota-Rojas et al. (22) have explained that vasodilation of the blood capillaries near the skin can increase the thermal exchange with the environment, which may explain the repercussions of modifying the thermal response at the surface level. Therefore, this explanation offers a partial understanding of the effects of local anesthetic administration.

Another possible explanation for this effect is that blockade of sympathetic fibers may lead to increased oxygen consumption in subcutaneous tissue (31). It has been reported that during acute pain perception, increased sympathetic tone causes vasoconstriction of blood capillaries, which may result in reduced oxygen delivery to the subcutaneous tissue (53). In contrast, Treschan et al. (54) have explained that increased parasympathetic tone can induce vasodilation, consequently increasing oxygen consumption in the subcutaneous tissue. In essence, blocking sympathetic motor fibers may enhance local parasympathetic tone, promoting vasodilation at the site and thereby increasing the availability of energy resources, such as oxygen. Consequently, this effect may facilitate heat radiation to the surrounding environment, which could explain the differences observed between both groups in this study. This could potentially be supported by what Lima et al. (55) reported, who observed that tissue oxygenation increased by 12% in the superficial tissue of the blockade site in healthy adult patients. This increase was likely due to local vasodilation and increased blood flow caused by the blockade of sympathetic nerve fibers. As a result of these processes, an increase in tissue oxygenation was observed. This coincides with the findings of Cañada–Soriano et al. (56), who observed that lumbar sympathetic blockade with lidocaine in patients with extremity pain caused an increase of 1.7 to 2.1 °C in the superficial temperature of the plantar region. However, in only 32% of the cases, no temperature change was recorded, which was considered indicative of a blockade failure. In animals, some authors have noted that injecting local anesthetics raised the superficial temperature at the injection site in horses, making it difficult to distinguish from other procedures, such as neurectomy (57, 58). The similarities in these findings suggest that changes in superficial temperature could indicate the effectiveness of the blockade, likely due to the suppression of the sympathetic response.

The control group exhibited a significant reduction in both surface and core body temperatures compared with baseline values at different time points. This phenomenon, commonly observed during general anesthesia, is partly attributed to the redistribution of body heat (59). Peripheral vasodilation induced by inhalant anesthetics initially decreases temperature in cooler regions, such as the skin, which subsequently promotes a gradual decline in central body temperature through a linear process, among other mechanisms (60). This mechanism may explain the differences observed between groups, as the PNB group demonstrated a significant reduction in Fe′Iso%. Moreover, several studies have suggested that the use of local anesthetic techniques in dogs exerts alveolar concentration-sparing effect (61–64). The observation that reductions in surface and core temperatures occurred only in the control group underscores the importance of balanced anesthesia strategies, including locoregional analgesia, to mitigate the risk of hypothermia.

Although vasodilation and superficial metabolic phenomena are the most widely accepted explanations for peripheral temperature changes, these phenomena can also be influenced by factors such as the use of cleansing solutions, inhalation anesthetics, or even trichotomy. The procedures are common in any surgery (65, 66). These findings are consistent with those reported by Wenham and Santos (67), who suggest that among the causes of decreased temperature are vasodilation from anesthetic agents, contact with cold solutions (like alcohol or antiseptic soaps), and hair removal around the surgical site. This highlights the critical need to consider the insulating properties of hair, which significantly attenuate superficial thermal responses. Previous reports indicate that hair can reduce surface temperature by approximately 1.5 °C by impeding convective and radiative heat exchange with the environment, an effect that is particularly pronounced in individuals with long hair (68). Evidence from both animal and human studies corroborates that hair removal enhances thermal dissipation through increased radiation (69, 70). Consequently, trimming hair may represent a necessary step to minimize this confounding variable. Conversely, antiseptic irrigation solutions applied during surgical procedures have been shown to produce a cooling effect on surface tissues (21). In support of this, Yilmaz et al. (71) demonstrated that irrigation of the surgical site resulted in a 1 °C decrease in surface temperature in a cohort of 32 New Zealand rabbits undergoing implant surgery. Taken together, these findings confirm that both hair coverage and antiseptic solutions directly modulate surface thermal dynamics, factors that may critically confound the objective evaluation of regional block efficacy. As a result, these factors can promote heat loss and potentially limit the accuracy of this study.

Although the results of the present study support the effect of local effects of anesthetics, this may be influenced by factors other than the effectiveness of the block. However, it is noteworthy that in the PNB group, total opioid consumption was significantly lower compared to the control group. Therefore, these findings may serve as indirect evidence of the success of the blockade. This emphasizes the importance of peripheral nerve blocks for managing acute pain (7, 9).

Finally, this study has several limitations that could be considered as areas for future research. This prospective clinical study was conducted under real hospital conditions, where group assignment was determined by clinical indication rather than randomization, reflecting ethical considerations in veterinary care. The treatment group (PNB) included dogs undergoing trauma-related surgeries requiring locoregional analgesia as standard care, resulting in a larger sample size. The control group comprised dogs undergoing general anesthesia for non-traumatic procedures without nerve blocks. This group was inherently smaller due to ethical constraints that prevent unnecessary surgical trauma in the absence of analgesia (72, 73).

Despite numerical disparities between the groups, the applied linear mixed-effects model appropriately accounts for unequal sample sizes and repeated measures, providing robust comparative analysis. Nonetheless, the limitation of unequal group sizes is acknowledged, and future studies should aim for larger, more balanced cohorts or internal controls to strengthen the findings. Therefore, future studies should compare the analgesic effectiveness of local anesthetics and continuous infusion analgesia. From a methodological perspective, the comparison of animals subjected to different surgical techniques represents a potential source of bias. As previously noted, hair can interfere with environmental heat dissipation, thereby further complicating the thermal assessment. This underscores the necessity of designing studies in which the local nerve block effect is evaluated under standardized surgical conditions. This approach would not only enhance the precision of comparisons but also address an essential statistical principle: all subjects should have an equal probability of selection and exposure to identical treatments (74).

Another key limitation is the absence of a comparison between the block’s effectiveness and other validated methods, such as ultrasound. Although ultrasound was used, no comparative study was conducted to validate its use. This presents an interesting avenue for further research, as it would be important to determine whether this technique could replace current methods or only serve as a supplementary approach. Another potential limitation is the limited variety of blocks selected and the small sample size of the sciatic nerve, which may affect the ability to verify the effects of local anesthetics at this dermal level. This highlights the need to explore whether similar responses occur with other types of nerve blocks and different thermal windows. Additionally, the limited evaluation times raise concerns since bupivacaine has a slow onset; evaluating thermographic variables within only 15 min leaves unanswered questions about whether the effects could extend beyond this period, such as 30 min or longer, and how they might correlate with analgesic outcomes. Furthermore, a significant limitation is the lack of an objective link between superficial thermal responses and the analgesic effectiveness of peripheral nerve blocks. Future research could focus on assessing the sensitivity, specificity, and reliability of this technique to determine critical cut-off points and evaluate the effectiveness of IRT through quantitative sensitivity testing. An additional avenue of investigation would be to determine whether this verification method could be applied to evaluate analgesic efficacy in traumatological surgical procedures. This approach could further enable the assessment of how concomitant pharmacological agents, including opioids such as fentanyl and inhalational anesthetics, modulate thermal responses and influence the interpretation of analgesic effectiveness.

## 5 Conclusion

The administration of 0.25% bupivacaine at 0.15 mL^−1^Kg^−1^ on the sciatic and saphenous nerves and 0.4 mL^−1^Kg^−1^ on the brachial plexus around peripheral nerves increases heat radiation compared to the control due to vasodilatation of the surrounding blood vessels. This effect may serve as an indirect indicator to assess the effectiveness of peripheral nerve blockade in dogs. However, it is important to consider future studies that confirm and validate its use for this purpose.

## Data Availability

The original contributions presented in the study are included in the article/supplementary material, further inquiries can be directed to the corresponding authors.
